# DNA immunization site determines the level of gene expression and the magnitude, but not the type of the induced immune response

**DOI:** 10.1371/journal.pone.0197902

**Published:** 2018-06-04

**Authors:** Stefan Petkov, Elizaveta Starodubova, Anastasia Latanova, Athina Kilpeläinen, Oleg Latyshev, Simons Svirskis, Britta Wahren, Francesca Chiodi, Ilya Gordeychuk, Maria Isaguliants

**Affiliations:** 1 Karolinska Institutet, Department of Microbiology, Tumor and Cell Biology, Stockholm, Sweden; 2 Engelhardt Institute of Molecular Biology, Russian Academy of Sciences, Moscow, Russia; 3 Chumakov Federal Scientific Center for Research and Development of Immune-and- Biological Products of the Russian Academy of Sciences, Moscow, Russia; 4 NF Gamaleja Research Center of Epidemiology and Microbiology, Moscow, Russia; 5 Riga Stradins University, Riga, Latvia; 6 Sechenov First Moscow State Medical University, Moscow, Russia; Imperial College London, UNITED KINGDOM

## Abstract

Optimization of DNA vaccine delivery improves the potency of the immune response and is crucial to clinical success. Here, we inquired how such optimization impacts the magnitude of the response, its specificity and type. BALB/c mice were DNA-immunized with two model immunogens, HIV-1 protease and reverse transcriptase by intramuscular or intradermal injections with electroporation. DNA immunogens were co-delivered with DNA encoding luciferase. Delivery and expression were monitored by in vivo bioluminescence imaging (BLI). The endpoint immune responses were assessed by IFN-γ/IL-2 FluoroSpot, multiparametric flow cytometry and antibody ELISA. Expression and immunogenicity were compared in relation to the delivery route. Regardless of the route, protease generated mainly IFN-γ, and reverse transcriptase, IL-2 and antibody response. BLI of mice immunized with protease- or reverse transcriptase/reporter plasmid mixtures, demonstrated significant loss of luminescence over time. The rate of decline of luminescence strongly correlated with the magnitude of immunogen-specific response, and depended on the immunogenicity profile and the immunization route. In vitro and in vivo BLI-based assays demonstrated that intradermal delivery strongly improved the immunogenicity of protease, and to a lesser extent, of reverse transcriptase. Immune response polarization and epitope hierarchy were not affected. Thus, by changing delivery/immunogen expression sites, it is possible to modulate the magnitude, but not the type or fine specificity of the induced immune response.

## Introduction

Plasmid DNA gained acceptance as a vaccine vehicle due to multiple advantages including an improved safety profile compared to live vaccines and viral vectors as well as relative simplicity of manipulation and production [1]. With the introduction of electroporation (EP)-assisted delivery, DNA uptake and gene expression were enhanced by up to 1000-fold, significantly improving the immunogenicity of this vaccine modality in animals larger than rodents [2, 3]. Series of studies characterized an additional adjuvant effect of EP linked to the local damage of the electroporated tissues that triggers the production of proinflammatory cytokines [4]. Multiple preclinical trials in both small and large animal models performed after the introduction of EP, demonstrated the capacity of DNA constructs to induce potent cellular and humoral host immune responses [5–7]. Altogether, this has turned DNA vaccination into a leading technique for the delivery of therapeutic and prophylactic vaccines against pathogens causing life-threatening acute and chronic human infections [8–11].

Much has been done in order to improve DNA vaccine delivery. Multiple new mechanical and non-mechanical methods have been introduced [12]. Most of these methods deliver DNA via intradermal (ID) or intramuscular (IM) administration. Which of these routes would be more preferable in the clinic in conjunction with EP has been heavily debated both in the context of the capacity of the anatomical sites to initiate and orchestrate the immune response, and due to the complexity, cost and ethical issues involved [5, 13–18].

A typical IM injection aims to deliver its load to skeletal muscle, which is unique in immunological terms. In physiological conditions skeletal muscle lacks antigen presenting cells (APCs) and has low levels of expression of major histocompatibility complex (MHC) I and II molecules. Inflammation in the skeletal muscles can upregulate expression of MHC I and II molecules turning myocytes into non-professional APCs [19]. This is often followed by activation of macrophages and accumulation of regulatory T cells, which favors tissue regeneration and suppresses immune response to minimize any possible autoimmune reactivities [20, 21]. Accumulation of CD4^+^ and CD8^+^ lymphocytes has also been reported, mostly due to the persistent inflammation or immune-mediated damage [22, 23]. Altogether, this suggests that immune intervention at this site prioritizes a timely termination of the inflammation process. On the other hand, the skin serves as the first line of immunological defense against infection [24] and both of its major compartments, epidermis and dermis, are abundantly populated with resident immune cells including dendritic cells (DCs), natural killer cells (NKs), CD4^+^ and CD8^+^ T cells [25–28]. The epidermis is also comprised of keratinocytes that play an important role in the immune response by releasing immune modulators [29, 30]. Thus, by administering an immunogen into the skin via an ID injection one could target multiple immune cell types and efficiently induce a potent multi-facetted immune response. The nature of the subsequent immune response would then be determined by the frequency and phenotype of APCs that encounter the antigen at the site of DNA inoculation [7], allowing to tailor the immune response to DNA vaccines by targeting it to different tissues (epidermis, dermis versus muscle).

HIV vaccines are in great need of improvement of both antibody and cellular responses to make them capable of preventing the infection and/or limiting viral load and replication in infected subjects [31, 32]. For the design of improved, likely multi-component, HIV DNA vaccines, it is crucial to understand if by choosing the delivery route, we can predefine the type of response they induce in the recipients, and also if the choice of a delivery route is going to affect the performance of different vaccine components in a similar way. To address these issues, we chose two model DNA immunogens derived from HIV-1: protease (PR) and reverse transcriptase (RT). In DNA immunization, PR induces potent cellular immune responses while generating no or very low humoral immune response [33]. RT is secreted by the expressing cells and as DNA immunogen induces an immune response characterized by high titer of IgG, induction of anti-RT IgA and secretion of interleukin (IL) 4 and 10 [34, 35]. This profile persists even after RT retargeting to the proteasomal route of degradation [35]. In the present study PR- and RT-based DNA immunogens were administered into skeletal muscles and skin by IM or ID needle injections, followed by EP. Plasmid delivery and expression were monitored by *in vivo* imaging of the luminescent signal emitted by the co-delivered firefly luciferase (Luc) reporter gene [36, 37]. We compared the effects of the immunization route (site of immunogen expression) on the type and magnitude of cellular and antibody responses. We show that ID delivery enhanced the immunogenicity of PR, and to a lesser extent of RT, but had no effect on either the type of immune response or the epitope dominance.

## Materials and methods

### Animals

The experiments were approved by the Northern Stockholm’s Unit of the Ethics of Animal Research on 16-05-2013 with ethical permit N66/13. The series of experiments were aimed at improving vaccines and vaccination strategies. Vaccine administration was allowed by intramuscular, intradermal, or subcutaneous routes to be performed using needle injections, inoculations with auxiliary devices, such as Biojector with or without electroporation. All pain inflicting procedures including injections, electroporations, and biojections were delivered under inhalation anesthesia, consisting of mixture of air and 2.5% isofluorane. The methods in these experiments were deemed to result in low degree of pain reflected by the no to little effect on normal weight, food and water consumption or behavior of the mice involved. Additionally, any possible mouse discomfort was alleviated by the application anesthesia. The animals were euthanized by cervical dislocation.

Eight-week-old, female BALB/c mice were purchased from Charles River Laboratories (Sandhofer, Germany) or from the breeding facility of the Department of Microbiology, Tumor and Cell Biology (Karolinska Institute, Stockholm, Sweden). Depending of the origin, animals were housed in Astrid Fagraeus Laboratory or in the animal facility of the Department of Microbiology, Tumor and Cell Biology under a light-dark cycle of 12 h / 12 h. Five to eight mice were contained in environment-enriched cages with food and water available *ad libitum*. All immunizations were performed using 29G needles and never exceeded the volume of 20 μL. Blood samples were acquired from the tail vein 2 and 4 weeks after immunization.

### Plasmid DNA

The luciferase-coding plasmid, pVax-luc 4663 bp (pVaxLuc) constructed by inserting the cDNA of firefly luciferase from pGL2-basic vector (Promega, # E1641) into vector pVAX1 (Invitrogen, # V260-20) under the control of a human cytomegalovirus immediate/early promoter and a polyadenylation signal from the bovine growth hormone gene [38], was kindly provided by Maltais AK (Karolinska Institutet, currently Eurocine Vaccines AB, Sweden).

Plasmids encoding RT variants were generated as described by us earlier [39]. In brief, amino acid sequence of RT of HIV-1 clade B HXB2 strain was encoded by a synthetic gene codon-optimized for expression in mammalian cells (Evrogen, Moscow, Russia). Coding sequence was supplemented with the Kozak sequence of inititation of translation. Resulting synthetic DNA was cloned into pVax1 vector (Invitrogen, USA) generating pVaxRTopt. Enzymatic activities were abrogated by site-directed mutagenesis, which introduced mutations D187N, D188N inactivating polymerase, and E480Q inactivating RNase H domains [34] generating plasmid pVaxRTopt-in. Loss of enzymatic activities was confirmed by *in vitro* tests [34].

The HIV protease (PR) plasmid was constructed by Hallengärd et al. by ligating a codon-optimized PR gene into a pKCMV vector. Mutations resulting in enzymatic inactivation (D25N) were introduced in the gene by site directed mutagenesis [33]. PR coding sequence was recloned into pVax1 vector using cloning scheme employed for HIV-1 RT generating pVaxPRin [39].

### Gene immunization and in vivo electroporation

Groups of 8-week old female BALB/c mice (n = 5 per experiment) were immunized with pVaxRTwt-opt, or pVaxRTwt-opt-in, or pVaxPRin mixed with pVax-Luc in 1:1 w/w ratio. Plasmids were injected in 20 μL of PBS in two sites to the left and to the right of the base of the tail using a 29G insulin syringe (Micro-Fine, BD, # 037-7614). Mice in the control group were immunized with pVax1 mixed with pVax-Luc. DNA injections were performed as was described previously [38]. Immediately after injection, the immunization sites were electroporated by placing a needle array electrode over the skin and applying 2 pulses of 1125 V/cm (50 μs interval) and 8 pulses of 275 V/cm (10 ms interval). The needle arrays consisted of eight 2-mm pins arranged in 2 rows (1.5 × 4 mm gaps) (BTX, # 47-0040). Electrical pulses were generated using the DermaVax system (Cellectis, Paris, France). The same electrode type was used to apply electrical current after both ID and IM injections. All experiments were performed as single immunizations where mice were injected with DNA and sacrificed 21 days later to assay the immune response. One experiment was performed as a prime-boost immunization. In this case, mice were primed as described above, and 28 days later boosted with the same plasmid mixture. Two weeks after the boost, mice were sacrificed to harvest spleens and blood for analysis.

### Real-time in vivo imaging

In vivo imaging of bioluminescence was performed with a highly sensitive CCD camera, mounted in a light-tight chamber (IVIS Spectrum CT, Perkin Elmer, Waltham, MA, USA) as previously described [37]. Anesthesia was induced by 4% isofluorane and maintained by 2.3% isofluorane throughout the imaging procedure. Ten minutes prior to capturing of the luminescent signal mice were injected intraperitoneally with the solution of D-luciferin (PerkinElmer, # 122796) in PBS at a dose of 150 mg/kg. The animals were monitored from the first time 2 h after immunization and then on days 1, 3, 9, 15, and 21(after prime). Before the acquisition of bioluminescence, selected mice were subjected to a microCT scan, delivering 23 mGy of radiation. Camera exposure time was automatically determined by the system and varied between 1–60 sec depending on the intensity of the bioluminescent signal. Regions of interest were localized around the injections sites and were quantified. CT/BLI data were processed using the Living Image software version 4.5 (Perkin Elmer) to generate values of signal intensity, depth, and volume of the transfected/ expressing area.

### IFN-γ and IL-2 FluoroSpot assay

Mice were sacrificed 21 days post prime, or 14 days post boost immunization, spleens were collected and cellular immune responses were measured by dual IFN-γ/IL-2 FluoroSpot assay according to the manufacturer’s instructions (MabTech AB, # FS-4142-10) as previously described [40]. All of the antibodies used in this assay were part of the commercialy available kit. Briefly, polyvinylidene difluoride plates were treated with 35% ethanol and coated with monoclonal antibodies for IFN-γ (AN18) and IL-2 (1A12) detection. A total of 2.5 × 10^5^ splenocytes per well were plated and stimulated for 20 h with antigens representing CD4^+^ and CD8^+^ T cells epitopes as well as full recombinant proteins or peptides at a concentration of 10 μg/ml. Refer to Table 1 for the amino acid sequences of all tested peptides. Cells from immunized mice were stimulated with a panel of peptides, representing known epitopes of HIV-1 1. All splenocytes were additionally tested for reactivity against the peptide GFQSMYTFV (GL Biochemical, Shanghai, China) representing a CTL epitope of luciferase recognized by BALB/c mice [41]. All peptides for splenocyte stimulation were used at a concentration of 10 μg/ml. The costimulatory anti-CD28 antibody was added to the cells during the incubation to stimulate production of IL-2 to synchronize it with secretion of INF-γ. Bound cytokines were detected with fluorescein isothiocyanate (FITC)-labeled INF-γantibody (R4-6A2) and biotynylated IL-2 antibody (5H4) followed by anti-FITC antibody conjugated to a green fluorochrome and streptavidin conjugated to a red fluorochrome. Finally, the number of spot-forming cells was detected using an iSpot reader (AID GmbH, Strassberg, Germany) allowing for the simultaneous analysis of cells secreting both cytokines.

**Table 1:** List of HIV-1 protease- (PR) and reverse transcriptase- (RT) derived peptides used for *in vitro* test of immune response. Experimentally-verified peptides present in the Los Alamos National Laboratory database are demarcated by an asterisk following their amino acid position.

Immunogen	Amino acid positions	Amino acid sequence	Reference
PR	1-15	PQVTLWQRPLVTIKI	[33]
PR	31-46	TVLEDINLPGKWKPKMIGGIGGFIKV	[33]
PR	56-70	VRQYDQILIEICGKK	[33]
PR	71-85	AIGTVLVGPTPVNII	[33]
PR	75-84*	VLVGPTPVNII	[33, 49, 50]
PR	76-90	LVGPTPVNIIGRNML	[33]
RT	63-72	IKKKDSTKWR	[51]
RT	66-76	KDSTKWRKLVD	[52]
RT	145-168	QYNVLPQGWKGSPSIFQSSMTKIL	[53]
RT	199-216	RTKIEELRTHLLRWGLTT	[53]
RT	205-220	LRQHLLRWGLTTPDKK	[53]
RT	207-215*	QHLLRWGLT	[54]
RT	207-223	QHLLRWGLTTPDKKHQK	[53]
RT	465-476	KVVPLTNTTNQK	[34, 55]
RT	514-528	ESELVNQIIEQLIKK	[56]
RT	528-543*	KEKVYLAWVPAHKGIG	[57]

### Intracellular cytokine staining and flow cytometric analysis

All of the reagents used for these tests were purchased from BD Biosciences (Franklin Lakes, NJ, USA) unless stated otherwise. Splenocytes from immunized or control mice (3 × 10^6^) were stimulated for 4-6 hr at 37°C and 5% CO_2_ with an equimolar mixture of peptides (10 μg/ml) representing known or predicted T cell epitopes. Concanavalin A (5 μg/ml) was used as a positive control. The stimuli were diluted in complete culture media consisting of RPMI supplemented with 5% FBS, 100 U/ml penicillin, 100 μg/ml streptomycin, and 0.3 mg/ml glutamine (Gibco, ThermoFisher, Waltham, MA, USA). Protein transport was inhibited by adding 1μg/ml of GolgiPlug to the stimulation mix.

To block unspecific binding of immunoglobulins to Fcγ receptors a CD16/CD32 antibody (cat. # 553142) was added to each well 10 minutes before the end of the incubation. Before proceeding to staining surface molecules, cells were stained for a viability using the Fixable Viability Stain 660 (FSV660, cat. # 564405) as recommended by the manufacturer. Surface staining was then performed by incubating the cells with a mixture of antibodies including: FITC-conjugated anti-mouse CD8a (cat. # 552877), APC-H7-conjugated anti-mouse CD4 (cat. # 560181) and PerCP-conjugated anti-mouse CD3 (cat. # 553067). Thereafter, the cells were fixed and permeabilized at room temperature for 20 minutes in 100 μL Cytofix/Cytoperm solution, washed with Perm/Wash buffer (cat. # 554714), and stained at 4°C for 30 minutes with PE-conjugated anti-mouse INF-γ(cat. # 554412), BV421-conjugated anti-mouse IL-2 (cat. # 562969) and BV510-conjugated anti-mouse TNF-α (cat. #563386) antibodies. The samples where then analyzed on a FACSVerse cytometer (BD Biosciences, Franklin Lakes, NJ, USA) and the data was exported as FCS3.0 files using the FACSuite software. The FCS files were subsequently read using the packages flowCore and openCyto in the R software language [42–44]. Finally, the cytometry data were normalized using the flowStats package and gated [45]. First a general lymphocyte area was defined and viable cells were identified by the lack for FSV660 staining. From the viable population, single cells were defined and that population was further gated according to the expression of surface markers, such as CD3, CD4, CD8 and the production of cytokines, such as IFN-γ, IL-2, TNF-α (S1 Fig).

### Humoral responses

Blood samples were drawn from the tail vein at the end-point of the experiment on day 21 post single, and on day 14 post prime-boost immunization. Serum samples were obtained by whole blood centrifugation and frozen at -20°C until thawed for ELISA. Plates were coated with 0.4 μg/ml recombinant RT or PR of HIV-1 clade B HXB2 strain (NIH AIDS Reagent program, Germantown, MD, USA) in PBS at 4°C overnight. Plates were washed and blocked with PBS containing 0.05% Tween 20, 0.5% of BSA and 1% goat serum for 1 h at 37°C. Diluted serum samples were then added and incubated at 4°C overnight. After washing, goat anti-mouse immunoglobulins conjugated to HRP (1:2000; Dako, Denmakr A/S, Glostrup, Denmark, # P0447) was added, and plates were incubated for 1 h at 37°C. Finally, plates were washed and developed with 3,3‘,5,5‘-tetramethylbenzidine (Medico-Diagnostic Laboratory, Moscow, Russia). After 15 min, the reaction was stopped by adding 50 μl of 2.5 M H_2_SO_4_ and the OD_450_ nm was determined [46]. Cut-off values were determined as average OD_450_ of sera from control vector immunized mice at dilutions 100 to 100 000 plus 3 STDV. End-point titer of PR- and RT gene immunized mice was calculated as the dilution at which OD_450_ of the serum sample fell below the cut-off.

## Results

### As model DNA immunogens, HIV-1 protease induces a CTL, and reverse transcriptase, an antibody immune response

**Fig 1:**
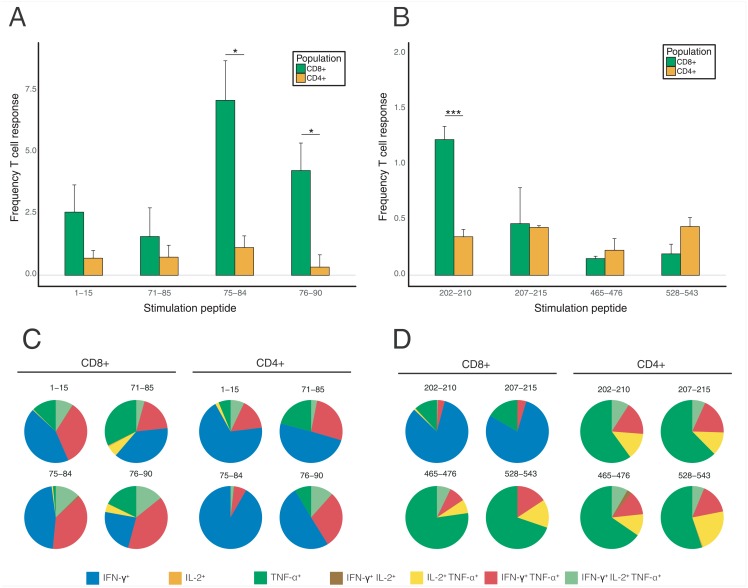
Analysis of protease and reverse transcriptase-specific immune response by flow cytometry. Analysis of CD4^+^ and CD8^+^ T cell responses in mice immunized with PR (A, C) and RT (B, D) presented as the percentage of the reactive CD8^+^ and CD4^+^ T cells (A, B) and as the percentage of cells producing INF-γ, IL-2, TNF-α, INF-γ/IL-2, INF-γ/TNF-α, IL-2/TNF-α, or INF-γ/IL-2/TNF-α with all reacting T cells taken for 100% (C, D). Peptides used for stimulation of murine splenocytes are designated by the number of first and last amino acid residues in the amino acid sequences of the respective proteins. The results of T cell response assays are from two to three independent DNA immunization experiments each done in 5 mice; all assays were done in duplicates. Frequency of T cell responses represents mean values ±SE. Statistical comparisons were done using multiple t-tests; *p < 0.05; **p < 0.01; ***p < 0.001; ****p < 0.0001.

We choose two HIV-1 clade B based DNA immunogens that generate different profiles of immune response: HIV-1 protease (PR), which induces potent cellular immune responses while generating no or very weak humoral immune response [33], and reverse transcriptase (RT), which as DNA immunogen induces an immune response characterized by high titer of specific IgG, induction of anti-RT IgA and low cellular immune response characterized by secretion of IL-4 and IL-10 [34, 35]. Before studying the relationship between expression and immunogenicity, we studied the specificity of the immune response elicited by these model immunogens defining recognized epitopes and phenotype of reacting T cells. To characterize the fine specificity of the response against these model DNA immunogens, we choose the ID route for delivery as it was shown to amplify the cellular response, revealing both dominant and subdominant epitopes [47, 48]. We immunized BALB/c mice by ID injections of pVax1-based plasmids carrying expression-optimized genes for PR and RT, with subsequent EP of the injection sites. Three weeks later, we collected spleens, isolated splenocytes, and stimulated them with PR- and RT-derived peptides representing established T cell epitopes of both immunogens (Table 1) [33, 35, 36]. Cytokine secretion of stimulated splenocytes was assessed by multiparametric flow cytometry probing production of INF-γ, IL-2 and TNF-α by CD4^+^ and CD8^+^ T cells (Fig 1, S1 Fig).

For PR, flow cytometry demonstrated a predominance of CD8^+^ T cell response targeted mainly against aa 75-84 (Fig 1A). By analyzing the response to adjacent peptides, we localized an additional epitope at PR aa 76-90 strongly recognized by the murine CD8^+^ T cells. Peptide recognition was manifested mainly by mono-secretion of INF-γ, to a lower extent, of TNF-α, their co-secretion (20-30%) and triple secretion of INF-γ/IL-2/TNF-α (5 to 15% of the reactive CD8^+^ T cells) (Fig 1C). Other peptides tested induced either low or no cellular response (Fig 1A, and data not shown).

CD4^+^ T cell responses were also present, but they were much less frequent, involving not more than 0.5% of the T cells (Fig 1A). The majority of the reactive CD4^+^ T cells (>50%) secreted only INF-γ, TNF-α (up to 25%) or TNF-α and IL-2 (10 to 20% of the reacting cells) (Fig 1C). In total, a PR-specific CTL response was induced in 15%, and T helper cell responses, in <3% of the respective T cell populations (Fig 1A), i.e. the response was predominantly cytolytic.

For RT, multi-parametric flow cytometry showed a modest cellular response by CD4^+^ and CD8^+^ T cells. We confirmed recognition in DNA immunized mice of one promiscuous CD8^+^ T cell epitope at aa 202-210 mapped earlier in the studies done in both mice, and humans [56, 57] (Fig 1B). The response against the promiscuous CD8^+^ T cell epitope at aa 202-210. This CTL epitope was manifested by the secretion of INF-γ, and infrequently, of INF-γ/TNF-α or INF-γ/IL-2/TNF-α (>75%), and <20% of the CD8^+^ T cells, respectively) (Fig 1D). The predominant response to two other weak CD8^+^ T cell epitopes was by mono-secretion of TNF-α (>75%) or co-secretion of TNF-α with IFN-γ or IL-2, or both (20-30% CD8^+^ T cells) (Fig 1D). The number of CD8^+^ T cells capable of dual or triple RT-specific cytokine secretion was the highest for the epitope at aa 528-543 (30% of the reactive CD8^+^ T cell population) (Fig 1D). Interestingly, and contrary to the PR-specific responses characterized by the predominant production of INF-γ, the prevailing response to CD4^+^ T cell epitopes of RT was by secretion of TNF-α alone (>60%), or together with IFN-γ or IL-2 (15-20% each), or of all three cytokines (10% of the responsive CD4^+^ T cell population) (Fig 1D). TNF-α was secreted by all RT-specific CD4^+^ T cells (over 1.5%) (Fig 1D).

Thus, multifunctional responses were induced in >50% of RT-specific CD4^+^ T cells (indicating their effector type/lytic character). In summary, RT-specific CTL responses were induced in 2,5% (six times lower than in the case of PR), and T helper cell responses, in 1.5% of the respective T cell populations (comparable to CD4+ T cell response against PR; Fig 1B), i.e. the response against PR was CTL-driven, and against RT, was largely balanced.

### Reporter gene co-delivery allows to monitor early immunogen expression in skin and muscle tissues

Building upon these data, we initiated a study of how the route of delivery of PR and RT encoding plasmids influences gene expression and the subsequent immune response. PR or RT were delivered into the murine skin by ID, or into the muscle tissues by IM syringe injections. All injections were followed by EP. To monitor the delivery, PR and RT DNA immunogens were mixed with a reporter plasmid encoding firefly luciferase (Luc). The delivery of plasmid mixtures, PR/Luc and RT/Luc, respectively, was monitored by *in vivo* bioluminescence imaging (BLI) that quantitatively assessed the expression of luciferase by Luc- and PR/Luc or RT/Luc co-transfected cells as compared to vector/Luc co-transfected cells in the control mice.

**Fig 2:**
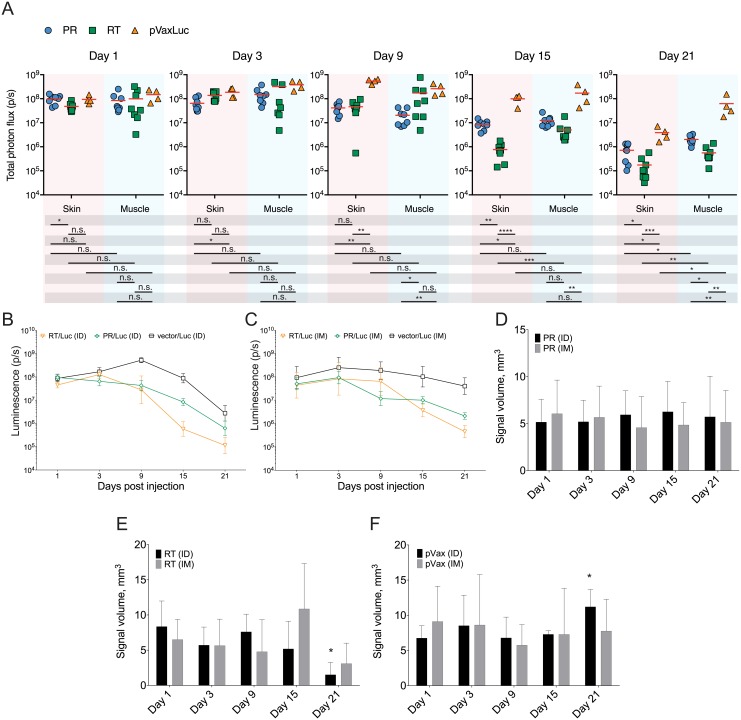
In vivo kinetics of reporter signal after co-delivery of luciferase, and HIV PR and RT genes. Delivery and expression of HIV-1 protease (PR) and reverse transcriptase (RT) encoded by their expression-optimized genes in pVax1 vector (PR, RT) monitored indirectly by their co-administration with a plasmid directing the expression of firefly luciferase pVaxLuc (Luc). Mice (n = 5 per group) were immunized by ID or IM injections of PR/Luc, or RT/Luc, or pVax1/Luc, 10*μ*g each plasmid, administered in PBS at two sites to the left and to the right from the base of the tail. Injections were followed by electroporation performed as described [58] using a DermaVax electroporator equipped with multi-needle electrodes (Cellectis, Paris, France). Total photon flux from the injection sites was assessed by BLI on days 1, 3, 9, 15 and 21 as described in the Materials and methods. Data represent individual values for each injection site, and mean values (A). Luminescence kinetics measured by 2D BLI after delivery of PR and RT by ID (B) and IM routes (C). Luminescence kinetics was also registered by 3D BLI demonstrating the volume of expressing tissues after PR/Luc (D), RT/Luc (E) and vector/Luc administration (F). Data represent average photon flux (photons/sq cm/sec) and expression volume (mm^3^) for 4 to 5 mice per group and time point, with two simultaneous measurements per mouse. Statistical comparison was done using Mann Whitney U-test; *p < 0.05; **p < 0.01; ***p < 0.001; ****p < 0.0001.

Three-dimensional bioluminescence tomography performed 2 and 24 hours post immunization assessed the quality of delivery in terms of the size and localization of the transfected area confirming skin and muscle localization of the expressing cells (S2 Fig and data not shown). The highest luminescence levels (in photons/sec, p/s) for mice receiving PR/Luc and RT/Luc equaled to 4.7 × 10^7^ p/s for ID and 9.9 × 10^7^ p/s for IM injections, and were reached during the first three days post plasmid administration, with a gradual decrease thereafter (Fig 2; S2 Fig panels A, B). At the early time points, skin and muscle supported similar levels of reporter expression. The intensity and dynamics of the reporter expression for PR/Luc and RT/Luc delivered IM and ID were similar up to day 3 (*p* > 0.05, Mann-Whitney test) (Fig 2; S2 Fig panels A-D). Thus, the addition of DNA immunogens had no influence on either the level, or early kinetics of the reporter expression, pointing at the usefulness of the method for assessing the overall quality of DNA delivery.

### Longitudinal kinetics of reporter expression is shaped by the expressing tissues, and by the nature of DNA immunogen

**Fig 3:**
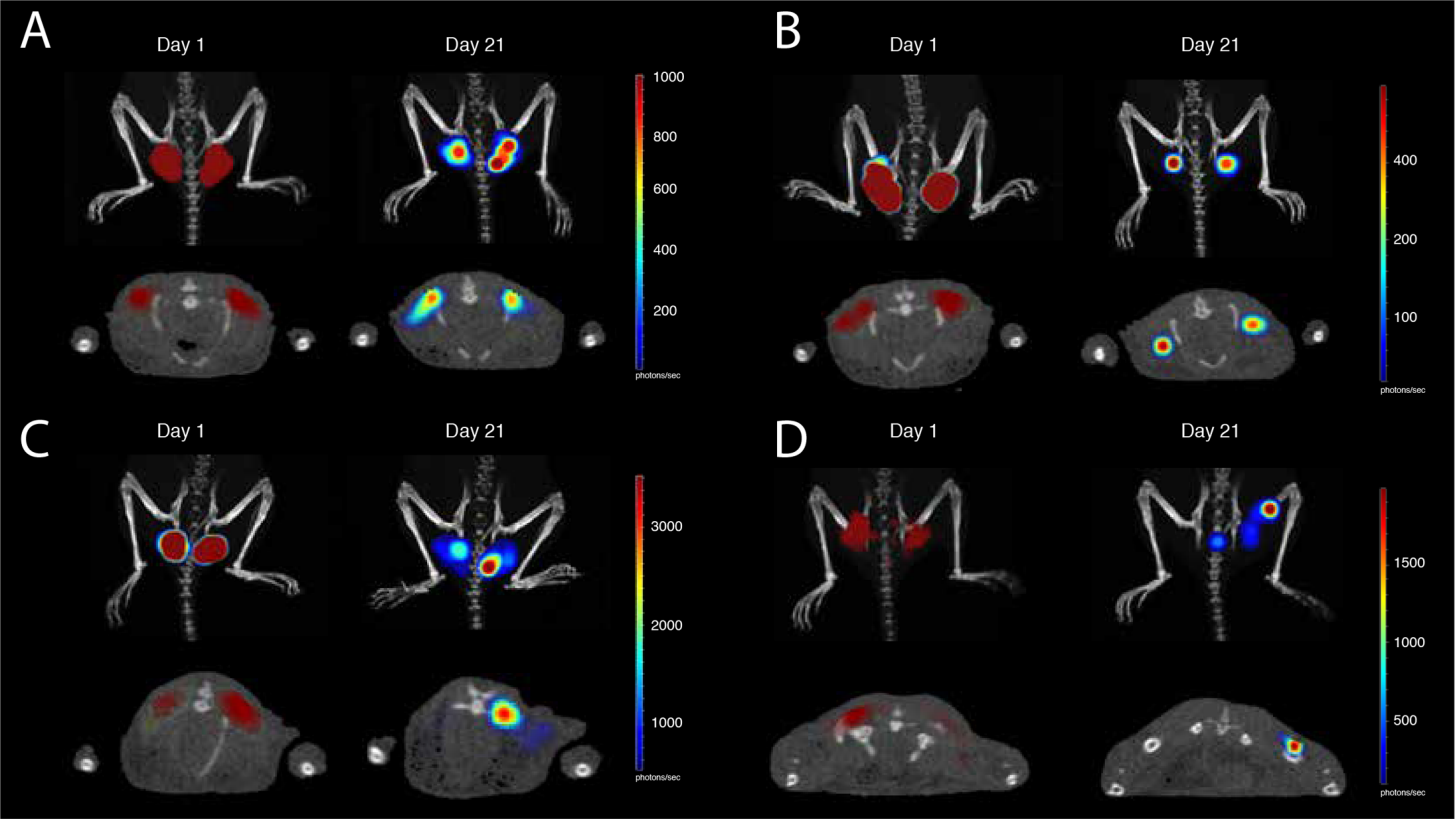
In vivo registration of the volume of tissues emitting bioluminescence. Volume of tissues emitting bioluminescence in mice immunized intradermally (A, B) or intramuscularly (C, D) with PR/Luc (A, C) or RT/Luc (B, D) with sequential imaging done 20-24 hours post injection (day 1) and by the experimental end-point (day 21) (as indicated by the text box over the panels). Images were acquired by combined 3D BLI and micro-CT on Spectrum CT with data analysis using Living Image 4.5 software (both Perkin Elmer). Panels A-D demonstrate one representative mouse in a group of five. Images of the upper row of panels A-D represent combined micro-CT and 3D BLI in the coronal section, and of the lower row, external illumination surface reconstruction. Signal intensity in photons/sec is represented as a color scale to the right of each image.

While the early expression of Luc reporter administered with PR and RT in the skin and in the muscle did not differ, we revealed significant differences in the reporter expression profiles from day 3 post injection and onwards. Luc expression directed by all mixtures (PR/Luc, RT/Luc and vector/Luc) decayed more robustly in the skin than in the muscle (Figs 2 and 3). The end-point levels of luminescence in mice receiving PR/Luc and RT/Luc DNA by the ID route were significantly lower than in mice receiving the same mixtures via IM. By day 21, the bioluminescence generated by PR/Luc- and RT/Luc delivered ID diminished by up to 800, and IM, by up to 500 times, 0.01 to 0.2% of the maximum, respectively (Fig 2). The signal at the sites of administration of vector/Luc DNA decreased significantly slower than for PR and RT mixtures, diminishing at the end-point 100 times for ID, and only 4 times for IM administrations. Of note, the maximal signal generated by vector/Luc DNA was reached at a later time point (day 3-9 vs day 1-3 for PR/Luc and RT/Luc mixtures) and was of a significantly greater intensity (Fig 2A–2C).

Further, we used 2D BLI to characterize the kinetics of bioluminescence loss for PR/Luc and RT/Luc administration. IM injection of PR/Luc DNA resulted in a distinct and abrupt loss of the reporter expression between days 3 and 9 (with much lower bioluminescence levels than in mice receiving PR/Luc intradermally; *p* = 0.002). The decrease at the later time points continued at a slower rate than for ID administration (Fig 2). As a result, at the experimental end-point, reporter expression in mice receiving PR/Luc ID was significantly lower than in mice receiving it by the IM route (5.0 × 10^5^ p/s compared to 3.0 × 10^6^ p/s; Fig 2A–2C; *p* < 0.05). On the contrary, the bioluminescence at the sites of IM and ID delivery of RT/Luc DNA demonstrated little change up to day 9, followed by a rapid decrease thereafter which continued until the last day of the follow-up. By the experimental endpoint, the bioluminescent signal generated by RT/Luc DNA was significantly lower than that for empty vector/Luc (0.9 × 10^5^ p/s compared to 5.0 × 10^6^ p/s for ID, and 5 × 10^5^ p/s compared to 1.0 × 10^8^ p/s for IM; Fig 2B and 2C, respectively). The rate and degree of signal loss from RT/Luc ID and IM injection sites were similar (although “fold-clearance” for the ID route tended to be higher than for IM (1.75 ± 1.85 × 10^5^ p/s for ID compared 5.62 ± 4.10 × 10^5^ p/s for IM; *p* = 0.007).

DNA immunization with RT, specifically ID, caused a more efficient decay of bioluminescence/reporter expression compared to immunization with PR. By day 15 of the follow up, signal from RT/Luc- and PR/Luc IM delivery sites decreased, but not significantly (*p* > 0.05; Fig 2C), whereas signal from the sites of ID delivery of RT decreased 10 times compared to that of PR (*p* < 0.01; Fig 2B). Furthermore, ID delivery culminated in significantly lower end-point values of bioluminescence at the RT- compared to the PR injections sites (0.9 × 10^5^ for RT/Luc DNA vs. 7.1 × 10^5^ for PR/Luc, *p* = 0.014).

We also performed 3D BLI with reconstruction of the volume of tissues expressing Luc after PR/Luc and RT/Luc administration in the skin and in the muscles. One day post-immunization, Luc expression was detected under the dermis (≥2 mm from the surface) after both ID and IM administrations, possibly due to EP with 2 mm long penetrating needle array electrodes that re-distributed plasmid into the deeper tissues (Fig 3A and 3B). After ID injections, the expression area was compact with an outer edge 2 mm below the level of the skin. In IM immunizations, expression penetrated even deeper, possibly due to the release of plasmid DNA into the blood capillaries with subsequent transfection of the cells in the downstream blood vessels. By day 21, expressing cells were still detectable as large diffuse volume of bioluminescent tissues (Fig 3C and 3D).

Sequential imaging demonstrated that after ID and IM deliveries of PR/Luc, the volume of expressing tissues (approx 5 mm^3^) did not change over time (*p* > 0.05; Figs 2D, 3A and 3C;). On the contrary, both ID and IM deliveries of RT/Luc DNA resulted in a significant reduction of the expression volume (from 5-10 to 1-2 mm^3^; *p* < 0.05 compared to PR/Luc and vector/Luc; Figs 3C and 3D and 2E). Reduction tended to be more pronounced for ID compared to IM delivery (*p* < 0.1; Figs 2E, 3C and 3D). As a result, by the experimental end-point, Luc expression at the sites of PR/Luc ID and IM immunization was observed as the area of the same size and localization as on day 1 but with a lower bioluminescence intensity. On the contrary, Luc expression at the sites of ID and IM RT/Luc immunization was seen as a greatly diminished area of weak luminescence in the deep tissues (Fig 3A and 3B). No reduction of the volume or average bioluminescence of expressing tissues was observed for mice receiving empty vector/Luc (Fig 2F).

Overall we found that administration of DNA immunogens into the skin resulted in a greater loss of the reporter signal over time compared to the muscle, regardless of the nature of immunogen encoded by co-delivered DNA. Further, we revealed that the kinetics, localization and the level of expression depended on the nature of the co-delivered DNA immunogen.

### Site of delivery defines the magnitude of but not the type of the response or epitope specificity

**Fig 4:**
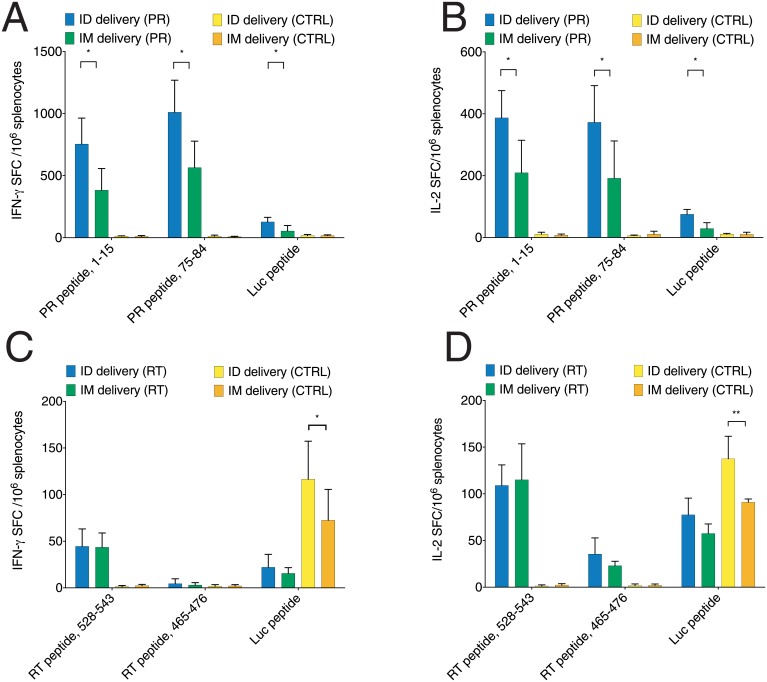
Cellular response against PR and RT induced by ID and IM DNA immunization assessed by FluoroSpot. Immune recognition of the peptides representing CD4^+^ and CD8^+^ epitopes of PR (A, B) and RT (C, D) by FluoroSpot test assessing in vitro production of IFN-γ (A, C) and IL-2 (B, D) in mice immunized with the plasmid encoding inactivated PR of HIV-1 HXB2 in pVax1 vector mixed with pVaxLuc (A, B); plasmid encoding inactivated RT of HIV-1 HXB2 in pVax1 vector mixed with pVaxLuc (C, D). Cytokine response to the immunodominant CTL epitope of Luc (LucP) in PR/Luc, RT/Luc and control empty vector/Luc DNA immunized mice (CTRL) is presented everywhere for comparison. Mice (n = 5 per group) were immunized as described in the legend to Fig 1 and their responses were assessed on experimental end-point at day 21 by INF-γ/IL-2 FluoroSpot. Data represent the average number of spot forming cells registered per million splenocyte per group (n = 5) with SE. Statistical comparison was done using Mann-Whitney U-test; *p < 0.05; **p < 0.01; ***p < 0.001; ****p < 0.0001.

Next, we characterized the immune response to PR and RT after intradermal and intramuscular DNA immunization by IFN-γ and IL-2 FluoroSpot. Murine splenocytes harvested 21 days post immunization, were stimulated *in vitro* with peptides that we have shown here to induce a multi-cytokine response, namely aa 1-15 and 75-84 of PR, and aa 465-476 and 528-543 of RT (Fig 1, Table 1). Splenocytes from the PR-immunized animals exhibited a clear divide in the readout. PR immunization resulted in the potent induction of cellular immunity both when delivered ID and IM. However, ID delivery generated much stronger cellular responses manifested by the production of IFN-γ and of IL-2 after stimulation with both peptide representing aa 1-15, and aa 75-84 of PR (Fig 4A and 4B). Interestingly, for mice immunized with RT/Luc DNA there was little difference in the magnitude or profile of cytokine production after ID and IM immunizations (n.s., Fig 4C and 4D). Both ID and IM immunization, induced IFN-γ and IL-2 response against the peptide representing RT aa 528-543, preferentially recognized by a population of murine CD4^+^ T cells (0.2% CD8^+^ and 0.5% CD4^+^; Fig 1C). The cellular response to the peptide representing RT aa 465-476, a CD8^+^ T cell epitope in humans [55] was low to non-existent (Fig 4C and 4D). The data may also reflect a propensity of FluoroSpot to capture IL-2 from the cell culture fluid depriving CD8^+^ T cells of the stimulation. The effect of IL-2 deprivation on Th2-polarized CD4^+^ T cells would be less pronounced, since they are stimulated not only by IL-2, but also by IL-4 [59]. Apart from this, anti-RT and anti-PR immune response induced by IM administration targeted same CD4^+^ and CD8^+^ T cell epitopes as those induced by the ID route. Cellular response to the CD8^+^ epitope of LucP was weak to insignificant after both ID, and IM delivery (Fig 4A and 4B).

**Fig 5:**
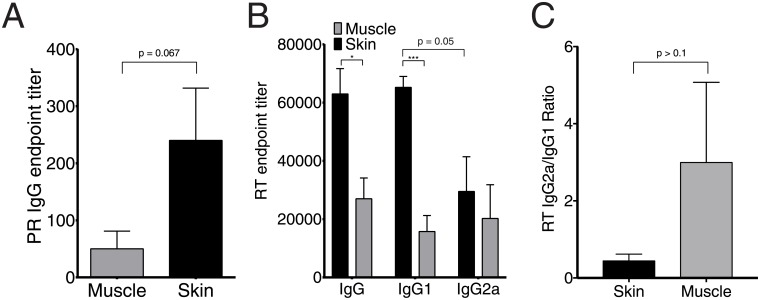
Antibody response against protease and reverse transcriptase assayed by ELISA. Antibody response in mice receiving PR and RT DNA by intradermal (skin) and intramuscular (muscle) delivery. Titer of IgG against PR (A), IgG, IgG1 and IgG2a against RT (B), and the ratio of anti-RT IgG2a/IgG1 (C). Mice (n = 5 per group) were immunized as described in the legend to Fig 1 and their responses were assessed on experimental end-point at day 21 by indirect ELISA on plates coated with recombinant PR (A), or RT (B, C) of HIV-1 HXB2 (NIH AIDS Reagent Program, Germantown, MD). Titer values represent average per group with SE. Statistical comparison was done using Mann Whitney U-test; *p < 0.05; **p < 0.01; ***p < 0.001; ****p < 0.0001.

As a CTL-inducing immunogen, PR elicited a very weak antibody response with titers less than 200 after ID, and no antibodies after IM immunization indicating a Th1-polarization (Fig 5A). In complete contrast, RT induced a strong antibody response. After single DNA-immunization, the titer of anti-RT IgG reached 2.7 ± 0.7 × 10^4^ in IM, and three-times higher levels (6.3 ± 0.8 × 10^4^) in ID immunization (Fig 5B). To see if the type of immune response induced by RT is affected by the route of delivery, we assayed the abundance of anti-RT antibodies of IgG1 and IgG2a subclasses, and calculated the IgG2a/IgG1 ratio, considering IgG2a/IgG1 <1 as an indication that an antigen induces a Th2-type immune response [60, 61]. ID immunization tended to give a stronger IgG1 compared to IgG2a response (*p* = 0.05, Fig 5B), whereas the levels of anti-RT IgG1 and IgG2a in mice receiving IM injections of RT/Luc DNA did not differ (*p* > 0.1). Due to this, IM administration yielded an average IgG2a/IgG1 ratio of more than 1, higher than that after ID administrations, however, the difference did not reach significance (Fig 5B and 5C).

Thus, for HIV-1 PR, immunization by IM route, induced only cellular response, and by ID route, enhanced cellular response and also low levels of specific antibodies. For HIV-1 RT, IM administration induced weak cellular and moderate antibody response; switching to ID route greatly enhanced the antibody arm of the response with no effect on the response polarization.

### Expression of co-delivered reporter gene as a surrogate in vivo marker of immune response against DNA immunogens

**Fig 6:**
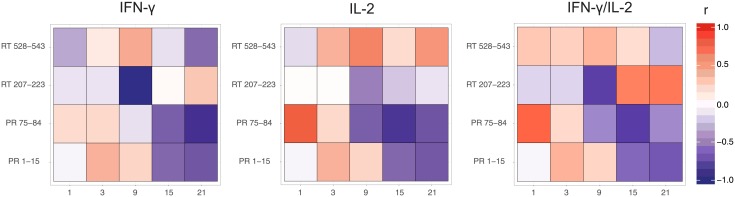
Correlation between bioluminescence of the reporter gene and specific cellular responses to co-injected DNA immunogens. Correlation of the fraction of luminescence signal retained at injection sites on days 1 to 21 post immunization to the number of splenocytes expressing INF-γ, IL-2 and co-expressing INF-γ/IL-2 in mice co-immunized with PR/Luc and RT/Luc (as described in legend to Fig 1). Strength of correlation is depicted by a scheme relating *r* values to color (red for direct, and blue for inverse) and its intensity (weak from 0 to 0.5, moderate 0.6-0.8, or strong, *r* > 0.8) on the right to the heatmap. Correlation values represent spearman’s rank correlation coefficients; *p < 0.05; **p < 0.01; ***p < 0.001; ****p < 0.0001.

We have previously shown that the loss of luminescence at the sites of reporter plasmid/DNA immunogen co-delivery correlates with the immunogen-specific cellular immune response manifested by the production of INF-γ [36, 37, 62]. To define this dependence for immunogens inducing cellular versus antibody responses, we juxtaposed the luminescent signal registered at every imaging time point with the antigen specific cellular and humoral immune response assayed at the experimental end-point. Anti-Luc cellular response had no impact on the loss of bioluminescence at the sites of co-delivery of DNA immunogens with Luc DNA and did not interfere with the correlation of luminescence with the immune response against co-delivered DNA immunogens (S2 Fig). Our analyses revealed significant correlations between the loss of bioluminescence and secreted INF-γ, IL-2 and INF-γ/IL-2 of PR-immunized mice in response to stimulation with peptides representing PR aa 1-15 and 71-84/75-84(Figs 1 and 6, S3 Fig). A highly significant inverse correlation of the intensity of luminescence with the frequency of T cells secreting IFN-γ in response to stimulation with peptides representing epitopes of PR aa 1-15 and 75-84 was observed starting from day 15 (*r* > 0.5, *p* < 0.05; S3 Fig panels A, B). Similar and even stronger correlations were identified for IL-2 and dual INF-γ/IL-2 response (Fig 6). On the contrary, RT DNA-immunized mice demonstrated no correlations of bioluminescent signal with the number of cells secreting INF-γ, IL-2 and INF-γ/IL-2 in response to *in vitro* stimulation with RT aa 465-476 and 528-543, which induce a multi-cytokine response of CD4^+^ and CD8^+^ T cells (*p* > 0.05; Fig 6).

Instead, we discovered a strong inverse correlation of the reporter signal with the magnitude of the end-point RT-specific antibody response (*r* = 0.7; *p* = 0.0006; S3 Fig panel E). Interestingly, however, we observed astrong inverse correlation of bioluminescence on day 9 with IFN-γ response induced by the peptide representing aa 207-223, which contains a cluster of CD4^+^ and CD8^+^ T cell epitopes (S3 Fig panel A). Furthermore, in mice receiving an RT DNA boost, we found a strong inverse correlation of bioluminescence with RT aa 207-223-specific production of IL-2 (S3 Fig panel A). Altogether, this confirms our earlier observation that the loss of bioluminescent signal manifests the immune clearance of antigen/reporter co-expressing cells.

## Discussion

In this study we addressed the two major vaccination routes in clinical use—ID and IM immunization. We analyzed the consequences of using each for DNA immunization by EP. To ensure high overall expression, we used a combination of two types of pulses: two 50 *μ*s-long high-voltage and eight 10 ms-long low-voltage pulses with amplitudes of 450 V and 110 V, respectively [2, 38, 58]. We delivered DNA immunogens into the skin and muscle tissues by needle injections, with accuracy confirmed earlier by 3D *in vivo* imaging of the immunization sites done within 2 h after plasmid administration [37], and confirmed the correct initial targeting by the differences in the kinetics of expression of reporter from a plasmid (Luc DNA), co-delivered with the target DNA immunogens. In ID delivery of immunogen/Luc DNA mixture followed by EP, the reporter expression peaked early and steadily declined thereafter. On the contrary, IM immunogen/Luc DNA delivery followed by EP led to a strong sustained reporter expression (Fig 2). These profiles corroborated our previous findings on the kinetics of Luc expression after ID and IM delivery of DNA encoding Luc alone [54] and were consistent with the expression kinetics described earlier for ID and IM DNA immunizations [63–65]. Interestingly, despite these differences in the expression profiles, and contrary to early time point observations [37], one day after DNA administration, we detected the expression of the reporter under the dermis in both ID and IM immunizations. We attributed this to the use of 2 mm long penetrating multi-needle electrode [37] that could have propelled plasmids deeper into the tissues from the sites of initial administration. Indeed, as was lately shown in the macaque model, the ID administration of DNA vaccines combined with EP extends antigen expression beyond the epidermis into the subcutaneous skin muscles [17]. Kos et al. have shown that in skin DNA administration, long pulses with low amplitude propel DNA into the muscle tissues, whereas short pulses of high amplitude constrain transfection and gene expression exclusively to the skin [66]. Our data obtained by *in vivo* 3D imaging confirms the results from these studies, suggesting as a target the panniculus carnosus muscle localized at a depth of over 2 mm [67]. Together with the findings of Todorova et al. these data demonstrate that ID DNA immunization with subsequent EP acts as a combination of the ID and IM immunizations ensuring the expression of immunogens by multiple cell types in all skin layers, as well as in subcutaneous and deep tissue structures, and due to this diversity possess a higher capacity for the development of potent immune response than IM immunization.

We further assessed how the route of delivery affected cellular and antibody responses elicited by different immunogens. For PR, ID immunization enhanced the cellular response and induced a weak antibody response, while delivery via the IM route, promoted only cellular responses. For RT, DNA immunization by ID enhanced, and by IM route, reduced the antibody response. Thus, ID administration potentiated the immune response against both immunogens by enhancing the responses characteristic to each. Importantly, the immune response induced against PR and RT by ID and IM immunizations involved the same epitopes. However, after IM administration, some of these responses (for example, against aa RT 465-476) were weak and difficult to detect. Indeed, muscle tissues are devoid of APCs as Langerhans cells or dermal dendritic cells. The immunogenicity relies on the infiltration of leukocytes attracted to the site of immunization by a variety of cytokines and chemokines [47] produced as a result of traumatization. On the contrary, ID administration targets dermal dendritic cells and macrophages. The immunogens are transported to the draining lymph nodes by activated resident APCs, monocytes or neutrophils (recruited to the site of inflammation from the peripheral circulation [47]) which in turn, prime local B- and T-cells initiating the adaptive immune response [68]. The latter route provides stronger stimulation, and results in a higher magnitude of the immune response. This may help reveal the subdominant epitopes, but does not seem to change the epitope hierarchy/dominance.

We have shown that the loss of bioluminescence from the sites of co-delivery of the Luc reporter with DNA immunogen inducing mainly cellular immune response, such as HIV-1 PR, correlated mainly with the activity of CTLs. However, when the immunogen induced mainly antibody response, such as HIV-1 RT, the titer of the specific anitbibodies exhibited a strong inverse correlation with the registered bioluminescence. Altogether, the loss of bioluminescent signal manifested the immune clearance of antigen/reporter co-expressing cells stressing the role of antibody responses in its clearance. Furthermore, we revealed that the administration of immunogen inducing a strong antibody immune response, HIV RT, both via ID and IM routes, resulted in a much lower end-point luminescence levels and volume of expressing tissues than the respective delivery of the immunogen inducing potent CTL response, HIV-1 PR. The bioluminescent signal from the sites of RT expression decayed at a higher overall rate than the signal from the sites of expression of PR.

The decay of the signal from PR- or RT/Luc co-expressing cells and cellular responses we observed were disperate. A superior PR-specific cell-mediated immunity was confirmed by all *in vitro* immunoassays, however, the end-point bioluminescent signal from the sites of RT/Luc co-expression as well as the end-point volume of RT/Luc co-expressing tissues were significantly lower than those for PR/Luc. Together with a strong correlation of RT/Luc-associated signal loss with the titer of anti-RT IgG, this indicated that the elimination of RT expressing cells involved alternative immune pathways. Antibody-dependent cellular cytotoxicity (ADCC) represents a possible mode of action that could explain the clearance of RT expressing cells. Series of studies stress the role of ADCC in controlling experimental HIV/SIV infections [69, 70]. Peptides derived from Pol polyprotein are common ADCC targets in HIV infection [71]. In rhesus macaques the induction of ADCC-promoting antibodies specific for CD4^+^-induced epitopes on the background of a balanced CD4^+^ T cell response correlated with protection from SIV infection. Our results showing that the ID DNA immunization with HIV-1 RT induces a strong antibody response associated with close to complete clearance of RT/reporter co-expressing cells from the sites of immunization corroborates the data generated by Fouts et al. in the macaque model [69]. Furthermore, in our case, as well as in their study, the antibody production was accompanied by a CD4^+^ T cell response against multiple epitopes within HIV RT, with the dominant one correlated to the *in vivo* clearance of RT/reporter expressing cells. The precise mechanism of immune clearance of RT-expressing cells needs to be further elucidated, but already at this stage, the lytic potential of the response points at the utility of this enzyme as a component of therapeutic HIV DNA vaccines.

In this study, RT DNA-immunized mice exhibited a multicytokine CD4^+^ T cell response characterized by the production of TNF-α, which is a marker of the effector/lytic activity. CD4^+^ T cells orchestrate the adaptive immune response against pathogens and regulate non-essential or deleterious activities. A growing body of evidence suggests that they possess a direct effector/cytolytic activity [72, 73]. We have described their lytic activity targeting cells expressing HIV-1 integrase [62]. A dominant role of CD4^+^ T cells in cell–mediated cytotoxicity/antigen clearance has also been demonstrated for a model DNA immunogen, SIV Gag-tagged luciferase [63]. When released, TNF-α binds to the TNF receptor-1, leading to the activation of an apoptotic cascade within multiple cell types [74] providing means for an efficient clearance of target cells. This cytokine profile could be another reason why we failed to detect a correlation between the clearance of luminescence and INF-γ/IL-2 production using FluoroSpot in case of RT DNA immunization. Alternative immune assays have to be applied to confirm correlations of reporter expression in RT/Luc immunized mice with anti-RT cellular response.

In conclusion, we have demonstrated by both *in vitro* and *in vivo* bioluminescence-based assays that ID delivery strongly enhances the magnitude of the immune response induced by model HIV-1 DNA immunogens that elicit celullar or antibody response. ID delivery “tailors” their immunogenicity in a similar way, enhancing the responses characteristic to each of the immunogen type, but not altering the response polarization, or the epitope hierarchy/dominance. Importantly, by intradermal DNA immunization followed by electroporation we were able to raise potent cellular and antibody responses against HIV-1 enzymes which were capable to clear the expressing cells from the sites of immunization. The magnitude of these responses and their effector capacity turn these immunogens into attractive components of therapeutic DNA vaccines.

## Supporting information

S1 Fig Identification of multifunctional T cell response. Debris and boundary events where first filtered out of the splenocyte population. Then only single cells were selected followed by a viability gating. The live lymphocyte population was then gated for CD4^+^ and CD8^+^ cells. Each individual population was then gated for INF-γ, IL-2 and TNF-α using tailgates and multiple cytokines by boolean gating.
(TIFF)

S2 Fig Correlation of bioluminescence emitted by the luciferase reporter and INF-γ response against the immunodominant epitope of luciferase GFQSMYTFV in the absence (A, B) and in the presence of co-delivered DNA immunogens (C, D). Correlation of the average radiance at the sites of injection of plasmid expressing Luciferase pVaxLuc alone to the number of splenocytes expressing IFN-γ in response to stimulation with GFQSMYTFV assessed by IFN-γ FluoroSpot on day 21 post immunization (*r* = −0.52; *p* = 0.07, Spearman rank correlation test) (A); Loss of the bioluminescence signal at the sites of ID administration of pVaxLuc mixed with the plasmid with no coding insert (B); encoding inactivated PR of HIV-1 HXB2 pVaxPR (C); encoding inactivated RT of HIV-1 HXB2 pVaxRT (D). In brief, mice (n = 5 per group) were immunized by two ID injections of respective plasmid mixtures followed by EP, as described in the legend of Fig 1. After 21 days, mice were sacrificed and immune response was assessed in splenocytes by INF-γ/IL-2 FluoroSpot. In panels B-D, the secretion of IFN-γ after *in vitro* stimulation of splenocytes with Luc peptide GFQSMYTFV was graded as >200 (designated as 200), 150-199 (150), 100-149 (100), 50-99 (50), or non-existing <50 (0) in terms of detected spot forming cells per million splenocytes, and given a symbol corresponding in size to the exhibited number of spots.
(TIFF)

S3 Fig Correlation between luminescence loss and IFN-γ responses against PR T cell epitopes 1-15 (A, days 15, 21) and 75-84 (B, days 15, 21). Only cellular response against an RT CD4^+^ T cell epitope 207-223 demonstrated a statistically significant correlation with luminescence after DNA prime (C; day 9) and after DNA boost (D; day 1). Sera from PR and RT immunized mice was obtained and analyzed for antibodies. Luminescence values were correlated with the level of RT-specific antibodies raised in mice by the experimental end-point on day 21 (E). Panels A, B, C, D each are constituted by three panels representing correlation analysis between the fraction of signal loss (x axis) and number of cytokine producing spots/cells after in vitro splenocyte stimulation with the peptides PR aa 1-15 (A), PR aa 75-84 (B); RT aa 207-223 (C, D) done on the days indicated over each of the sub-panels.
(TIFF)
