# Correction: DNA immunization site determines the level of gene expression and the magnitude, but not the type of the induced immune response

**DOI:** 10.1371/journal.pone.0247239

**Published:** 2021-02-11

**Authors:** Stefan Petkov, Elizaveta Starodubova, Anastasia Latanova, Athina Kilpeläinen, Oleg Latyshev, Simons Svirskis, Britta Wahren, Francesca Chiodi, Ilya Gordeychuk, Maria Isaguliants

There are errors in the Funding statement. The grant number listed from the Russian Foundation of Basic Research is actually from the Russian Science Foundation. The correct Funding statement is as follows: The study was supported by the Russian Foundation of Basic Research project #17_04_00583 (http://www.rfbr.ru/rffi/ru/) and by the Russian Science Foundation project #15-15-30039. Cross-border partner interactions were supported by the EU Twinning project VACTRAIN #692293 and PI project of the Swedish Institute #19806_2016. Participation of Petkov S, Chiodi F, and Wahren B was supported by VACTRAIN. Participation of Svirskis S was supported by BALTINFECT.

The authors also incorrectly declared that no competing interests exist. The correct Competing Interests statement is as follows: The authors have read the journal’s policy and the authors of this manuscript have the following Competing Interests: Dr Kajsa Prokopec, Dr Jens Gertow, Dr Niklas Ahlborg and all the staff of the Research and Development Department of Mabtech, Nacka, Sweden, provided continuous support with FluoroSpot applications, assistance in the analysis of the data and comments on the manuscript. This does not alter our adherence to PLOS ONE policies on sharing data and materials.

S1 Dataset is omitted from the list of Supporting Information. It can be viewed below.

## Supporting information

S1 Dataset This file includes supplementary data. (XLSX)
