# Supplementary figures and images for: DNA immunization site determines the level of gene expression and the magnitude, but not the type of the induced immune response
Source: PLoS One. 2018 Jun 4;13(6):e0197902. doi: 10.1371/journal.pone.0197902 (PMC5986124; doi:10.1371/journal.pone.0197902)

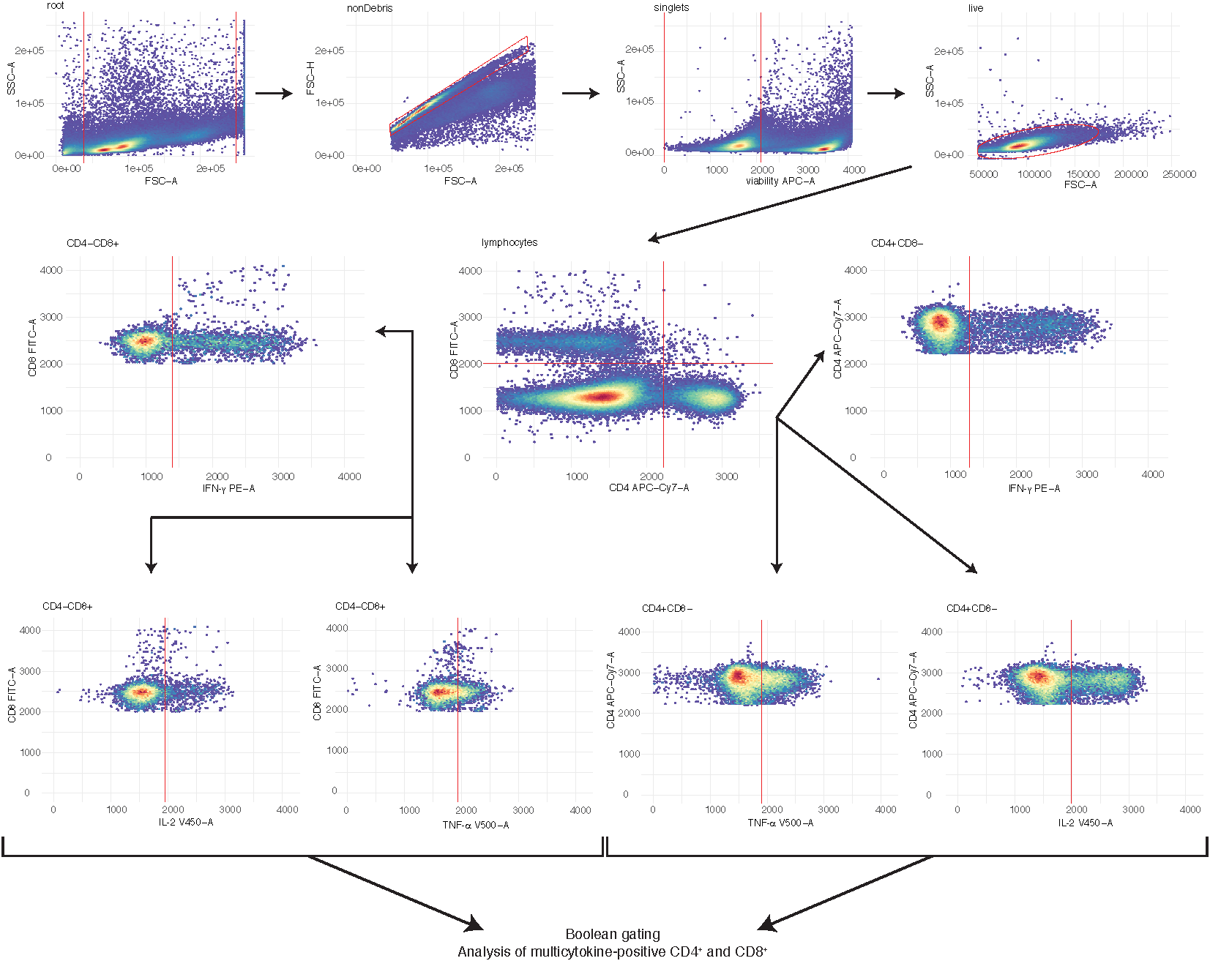

Supplement: S1 Fig — Debris and boundary events where first filtered out of the splenocyte population. Then only single cells were selected followed by a viability gating. The live lymphocyte population was then gated for CD4+ and CD8+ cells. Each individual population was then gated for INF-γ, IL-2 and TNF-α using tailgates and multiple cytokines by boolean gating. (TIFF) [file pone.0197902.s001.tiff]

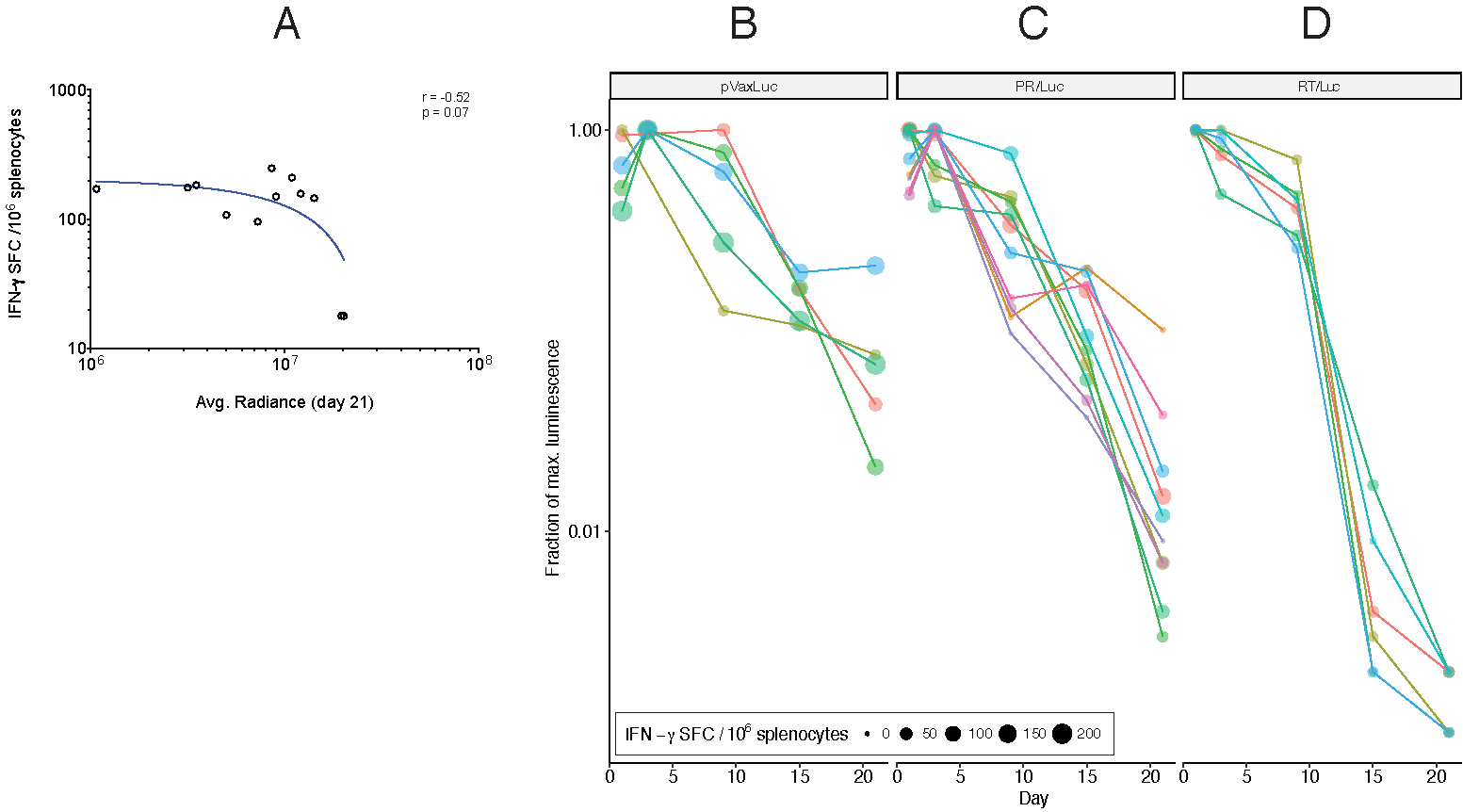

Supplement: S2 Fig — Correlation of the average radiance at the sites of injection of plasmid expressing Luciferase pVaxLuc alone to the number of splenocytes expressing IFN-γ in response to stimulation with GFQSMYTFV assessed by IFN-γ FluoroSpot on day 21 post immunization (r = −0.52; p = 0.07, Spearman rank correlation test) (A); Loss of the bioluminescence signal at the sites of ID administration of pVaxLuc mixed with the plasmid with no coding insert (B); encoding inactivated PR of HIV-1 HXB2 pVaxPR (C); encoding inactivated RT of HIV-1 HXB2 pVaxRT (D). In brief, mice (n = 5 per group) were immunized by two ID injections of respective plasmid mixtures followed by EP, as described in the legend of Fig 1. After 21 days, mice were sacrificed and immune response was assessed in splenocytes by INF-γ/IL-2 FluoroSpot. In panels B-D, the secretion of IFN-γ after in vitro stimulation of splenocytes with Luc peptide GFQSMYTFV was graded as >200 (designated as 200), 150-199 (150), 100-149 (100), 50-99 (50), or non-existing <50 (0) in terms of detected spot forming cells per million splenocytes, and given a symbol corresponding in size to the exhibited number of spots. (TIFF) [file pone.0197902.s002.tiff]

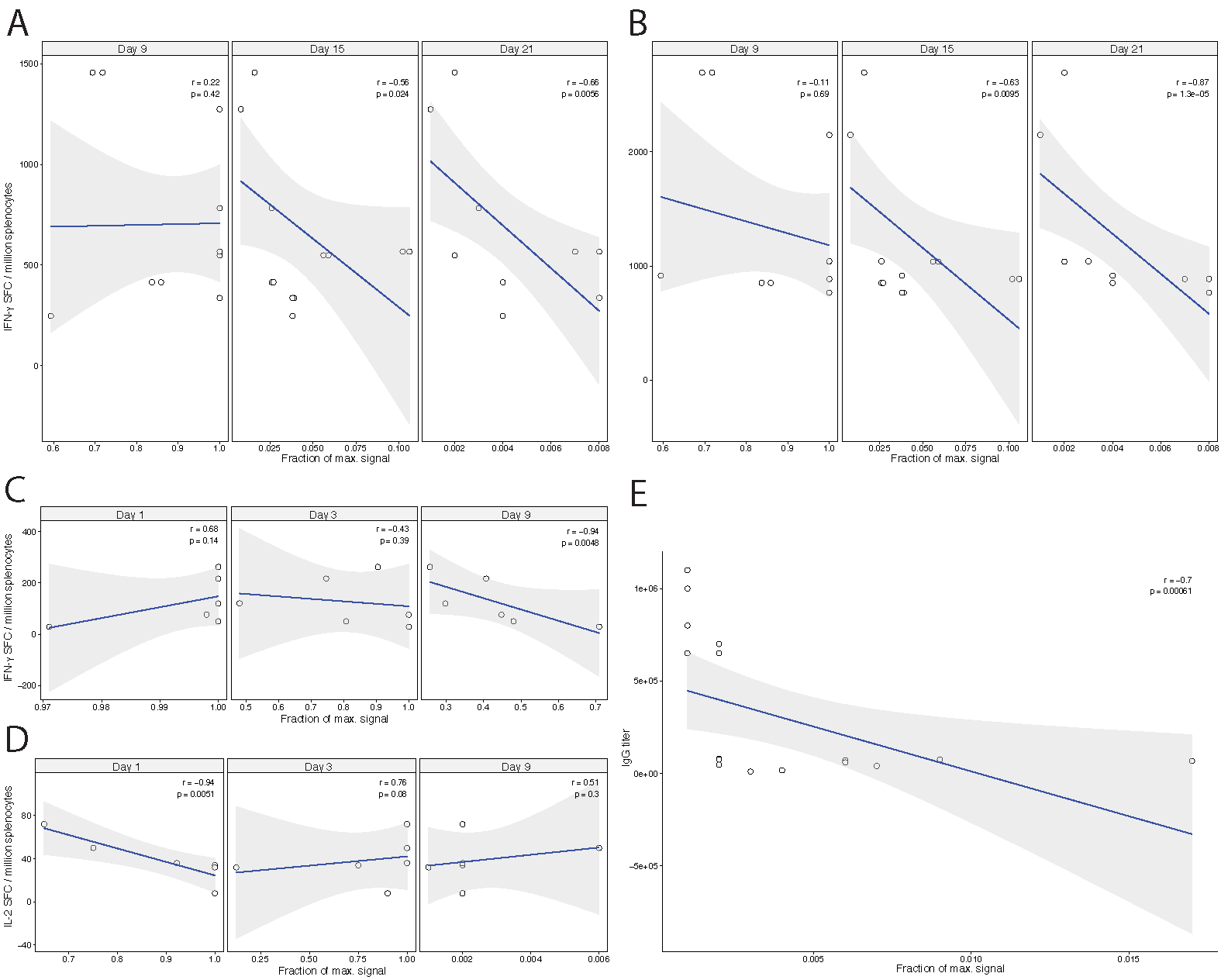

Supplement: S3 Fig — Correlation between luminescence loss and IFN-γ responses against PR T cell epitopes 1-15 (A, days 15, 21) and 75-84 (B, days 15, 21). Only cellular response against an RT CD4+ T cell epitope 207-223 demonstrated a statistically significant correlation with luminescence after DNA prime (C; day 9) and after DNA boost (D; day 1). Sera from PR and RT immunized mice was obtained and analyzed for antibodies. Luminescence values were correlated with the level of RT-specific antibodies raised in mice by the experimental end-point on day 21 (E). Panels A, B, C, D each are constituted by three panels representing correlation analysis between the fraction of signal loss (x axis) and number of cytokine producing spots/cells after in vitro splenocyte stimulation with the peptides PR aa 1-15 (A), PR aa 75-84 (B); RT aa 207-223 (C, D) done on the days indicated over each of the sub-panels. (TIFF) [file pone.0197902.s003.tiff]
